# Prevalence of Malnutrition and Depression in Older Adults Living in Nursing Homes in Mexico City

**DOI:** 10.3390/nu12082429

**Published:** 2020-08-13

**Authors:** María Consuelo Velázquez-Alva, María Esther Irigoyen-Camacho, María Fernanda Cabrer-Rosales, Irina Lazarevich, Isabel Arrieta-Cruz, Roger Gutiérrez-Juárez, Marco Antonio Zepeda-Zepeda

**Affiliations:** 1 Health Care Department, Metropolitan Autonomous University, Unit Xochimilco, Mexico City 04960, Mexico; mcvelaz@correo.xoc.uam.mx (M.C.V.-A.); mcabrer@correo.xoc.uam.mx (M.F.C.-R.); iboris@correo.xoc.uam.mx (I.L.); mzepeda@correo.xoc.uam.mx (M.A.Z.-Z.); 2 Department of Basic Research, National Institute of Geriatrics, Ministry of Health, Mexico City 10200, Mexico; iarrieta@inger.gob.mx; 3 Department of Biomedical Sciences, School of Medicine, Faculty of High Studies Zaragoza, National Autonomous University of Mexico, Mexico City 09230, Mexico; roger.gutierrez@zaragoza.unam.mx

**Keywords:** malnutrition, depression, older adults, institutionalized, nursing home residents

## Abstract

This study evaluated the association between nutritional status, depressive symptoms, and the number of prescription drugs taken by older adults living in nursing homes in Mexico City. In a cross-sectional study, 262 participants were subjected to anthropometric and nutritional (Mini Nutritional Assessment (MNA)) evaluations; additionally, their depression (Geriatric Depression Scale (GDS)) and functional status were assessed. Multiple logistic regression was used for identifying factors associated with the risk of malnutrition/malnourishment. The mean age of participants was 83.1 ± 8.6 years. A total of 59.9% and 21.1% were at risk of malnutrition and malnourished, respectively. With respect to depression, 27.9% of the participants had mild depression, while 11.4% showed severe depression. An inverse correlation between MNA evaluations and depression scores was found (Spearman’s *ρ* = −0.4624, *p* < 0.001); residents with a better nutritional status had lower depression scores. Individuals with depressive symptoms were approximately five times more likely to be at risk of malnutrition or malnourished (OR = 5.82, 95% CI = 2.27–14.89) than individuals without depression. Residents taking three or more prescription drugs daily (OR = 1.83, 95% CI = 1.27–2.63, *p* < 0.001) were more likely to be at risk of malnutrition or malnourished. In summary, poor nutritional status was associated with depression, while the intake of numerous prescription drugs was associated with being at risk of malnutrition or malnourished.

## 1. Introduction

Nutrition is one of the most important determinants for the overall health of nursing home residents, where malnutrition is a common finding. Malnutrition during aging has a multifactorial etiology and may severely influence the health status of older adults [1]. Malnutrition is a chronic and frequently imperceptible process, and its identification at the early stages is of the utmost importance for reducing adverse outcomes, such as increased dependency, compromised quality of life, higher costs of medical care, and increased morbidity and mortality rates [2].

A recent meta-analysis study including cross-sectional, prospective, retrospective, and case-control studies showed that the prevalence of malnutrition is quite different in older adults living in the community (4.0%) compared to those who are hospitalized (22.3%), those who are living in nursing homes (15.2%), or those in long-term care institutions (30.0%) [3]. In this regard, very little information is available about the malnutrition prevalence in older Mexican persons, either community-dwelling or living in nursing homes [4,5].

Older adults suffer multiple chronic conditions and their emotional health is frequently affected by feelings of depression, such as sadness, misfortune, and unhappiness, leading to broken spirits. Depression is a potentially disabling psychiatric disorder that is commonly associated with an increase in mortality rate [6]. Older people in nursing homes have a higher risk of depression than non-institutionalized ones [7,8]. The prevalence of depressive symptoms varies considerably around the world; for example, in a systematic review including mainly observational studies in long-term care homes from several countries showed that the median prevalence of depressive symptoms was 29.0%, ranging from 14% in the US to 82% in southern Taiwan [9]. Despite this, the residents of many geriatric institutions remain undiagnosed and untreated, thus negatively affecting their quality of life and well-being [10]. Another retrospective review study indicated that depression is one of the main factors associated with weight loss in nursing home residents [11]. Additionally, a cross-sectional study in hospitalized older adults identified an association between depression and undernutrition [12].

Furthermore, the multiple chronic conditions affecting older people quite often require the simultaneous use of several prescription drugs for treatment. The groups of drugs more frequently used by older patients are directed to the cardiovascular and nervous systems, digestive tract, and metabolism [13]; thus, multiple comorbidities encourage the use of polypharmacy. In a study involving Japanese older adults in nursing homes, the mean number of prescription drugs taken per patient was 5.9 [14], while in a population of Finnish older women and men, the mean number was 8.2 and 7.7, respectively [15]. Polypharmacy increases the risk of drug interactions, treatment cost, and risk of side effects. As expected, older adults living in residential homes are more likely to be on a higher number of drugs than outpatients [14].

A few studies have investigated the association between nutritional status and polypharmacy; however, their findings are conflicting [16,17]. For example, a cross-sectional study in a community-dwelling sample of older people in Spain found that polypharmacy was associated with malnutrition /risk of malnutrition [18]. In contrast, another cross-sectional study conducted on Italian nursing home residents with severe functional impairment did not find an association between the number of drugs taken and nutritional status [17]. Additionally, the use of multiple definitions of polypharmacy in the literature makes it difficult to compare the results of the various studies. The synergistic relationship between malnutrition and depression is potentially negative for survival, and consequently, the quality of life may worsen when inadequate monitoring of medication prescription/administration is being performed. Additional information from different groups of older people is required not only to determine the extent of the relationship between these factors but also to design interventions targeting the key factors affecting the health status of older adults and to promote strategies to improve their quality of life. Thus, the aim of this study was to examine the association between nutritional status, depressive symptoms, and the number of prescription drugs used in a group of nursing home residents from Mexico City.

## 2. Materials and Methods 

### 2.1. Study Design

A cross-sectional study was conducted in three private nursing homes located in the south-eastern area of Mexico City. A total of 337 residents were distributed in these three residences as follows: 100, 116, and 121 older adults, respectively. Middle-income older adults paying around one hundred and twenty US dollars a month reside in these nursing homes, which were managed by a private assistance non-profit organization. The study was carried out from May to October 2018. This study was in accordance with the ethical standards of the Declaration of Helsinki. The protocol was registered by the Council of the Division of Biological Sciences and Health of the Metropolitan Autonomous University and approved by the Institutional Ethics Committee (code: DCBS.CD, approval CD.52.17).

The objectives of the study and procedures involved were explained to each participant, and a signed written consent form was obtained from each of them. The inclusion criteria were: men or women, 65 years and older, staying for more than one year in the nursing home, capable of answering the questionnaire by him/herself, and providing written consent. Only five older adults were unwilling to participate in the study, resulting in a participation response rate of 98.5%.

The Charlson Index, Cumulative Illness Rating Scale (CIRS) was used to establish the inclusion and exclusion criteria for the participants, where the criteria are based on the information obtained from the medical records in the file of each patient [19].

Older adults with cognitive impairment or dementia, bedridden, functionally dependent, severe dysphagia, chronic obstructive pulmonary disorder (COPD), and end-stage illnesses were not included since these patients usually have a pre-existing history of weight loss, decreased body mass index, or deteriorated nutritional status and a higher risk of infections [20,21,22,23,24,25,26].

Based on the residents’ medical history, 70 older adults were not included because 47 of them had cognitive impairment or dementia in advanced stages, while the remaining 23 had one or more of the medical conditions mentioned above. Thus, data from only 262 participants were analyzed.

The following sociodemographic information was used from the medical records of the participants: date of birth and admission to the nursing home, educational attainment, pre-existing medical conditions, use of prescription drugs, and current antidepressant use (within the past 30 days).

Two certified dieticians performed the anthropometry. The standardization procedures were conducted by a certified dietitian [27,28]. During the study, 25 participants (9.5%) were re-evaluated independently to assess the examiner’s reliability. The results indicated a 90% inter-examiner agreement regarding the anthropometric information.

#### 2.1.1. Nutritional Status Assessment

The anthropometric evaluation considered the following measurements: weight, height, circumferences (calf (CC), mid-upper-arm (MUAC), arm muscle (AMC)), and tricipital skinfold (TSF) [29]. The cut-off values of the World Health Organization (WHO), Geneva, Switzerland, and Lipschitz classification were used to identify underweight [30,31].

To evaluate the nutritional status, the Mini Nutritional Assessment (MNA) was used. Older adults with a score <17 were classified as malnourished, those with an MNA score of 17–23.5 were considered at risk of malnutrition, while older people with an MNA > 23.5 were classified as well-nourished [32].

#### 2.1.2. Functional Status

The Barthel Index (BI) was used to evaluate the activities of daily living (ADL). This index, ranging from 0 to 100, is a scale for estimating disability or dependence in ADL in geriatric patients (feeding, bathing, dressing, grooming, bowels and bladder (incontinence), toilet use, transfers (bed to chair and back), walking with the help of a person, going up/down the stairs (needs help)). A score of ≤60 indicated the presence of physical disability [33].

#### 2.1.3. Depression Assessment

The presence of depressive symptoms was assessed using the Geriatric Depression Scale (GDS). This scale ranges from 0 to 15, with a higher score indicating depression. The cut-off points used for the classification of depression were as follows: 0–5 normal, 6–10 mild depression, and ≥10 established depression [34].

The results were disclosed to the participants and the personnel responsible for the nursing home medical services.

### 2.2. Statistical Analysis

The data are presented as means ± standard deviations for continuous variables and percentages for categorical variables. Correlation analysis was performed using Spearman’s *ρ* correlation coefficient, owing to the non-normal distribution of the variables. Pearson’s *χ*^2^ test was used for comparison of the categorical variables.

The MNA score was used to classify older persons into two groups: (1) at risk of malnutrition, and (2) malnourished, both of which were assigned to a single group, and the well-nourished residents were assigned to another group. Logistic regression models were constructed to determine nutritional status. Crude, adjusted odds ratios (ORs), and 95% confidence intervals (95% CIs) were obtained; considering that the participants were living in three different nursing homes, the analysis was performed using the cluster option and robust standard estimators were obtained. The goodness of fit of the model was evaluated using Hosmer–Lemeshow’s test. Interactions between sex, depressive symptoms, and the number of prescription drugs were explored in the multiple logistic regression model. Statistical significance was set at *p* < 0.05. The statistical package STATA V15 (Stata Corporation, College Station, TX, USA) was used for the data analysis.

## 3. Results

A total of 262 older adults were included in the study: 88 men and 174 women. The mean age was 83.1 ± 8.6 years. No significant differences in age, sex, anthropometry, and MNA were found between the three nursing homes included. Table 1 presents the demographic, socioeconomic, clinical, and nutritional characteristics of the study group by sex. The mean number of years living in the nursing homes was 4.1 ± 4.4. The most prevalent chronic diseases were hypertension 58.4% (*n* = 153), type 2 diabetes mellitus 22.5% (*n* = 59), and cardiovascular diseases 17.6% (*n* = 46), while 20.2% (*n* = 53) were diagnosed with depression. A total of 37 (14.0%) patients were taking antidepressants, while the most frequently used medications were sertraline, fluoxetine, and paroxetine.

Regarding the number of prescription drugs taken by the participants, an average of 5.1 ± 3.9 drugs/24 h was observed, with no significant difference observed when stratified by sex. The percentage of participants taking three or more drugs was 73.7% (*n* = 193), while those taking five or more drugs (polypharmacy) accounted for 46.2% (*n* = 121) of the total.

BMI determinations using the WHO criterion (BMI < 18.5 kg/m^2^) showed that 10.7% of the participants were underweight. Instead, when the Lipschitz criterion (BMI < 22 kg/m^2^) was applied, more than one-third (34.4%) of the participants were classified as underweight. Significant differences in the anthropometric values by sex were observed only in height and TSF (Table 1).

The MNA results indicated that only a low percentage (17.9%) of participants were well nourished. No significant differences between males and females were observed. The GDS data showed statistical differences between men and women (4.1 ± 3.0 vs 5.1 ± 3.6; *p* = 0.0275). Estimations of the depression level of the study subjects showed that 39.3% (*n* = 103) of them had mild (27.9%) or severe (11.4%) depression (Table 1). Interestingly, severe depression was significantly more frequent in women than in men (*p* < 0.001), as shown in Table 1.

The BI mean score of the study population was 65.0 ± 35.0, with clear differences between men and women (69.7 ± 32.3 vs. 62.5 ± 36.1, respectively; *p* = 0.0133). No significant differences were found in the dependency levels by sex. When the results were grouped by nutritional status, statistical differences were found for the BI and some anthropometric measurements (Table 2).

For depression, the results were dichotomized into non-depression and mild-and-severe depression groups. It was found that some anthropometric measurements, such as calf circumference, MUAC, and TSF were significantly different between these depression categories (*p* < 0.001). Furthermore, the BI and MNA were significantly associated with depression (Table 3). There was no relationship between the use of anti-depressant drugs and nutritional status (men *p* = 0.143, women *p* = 0.416).

**Figure 1:**
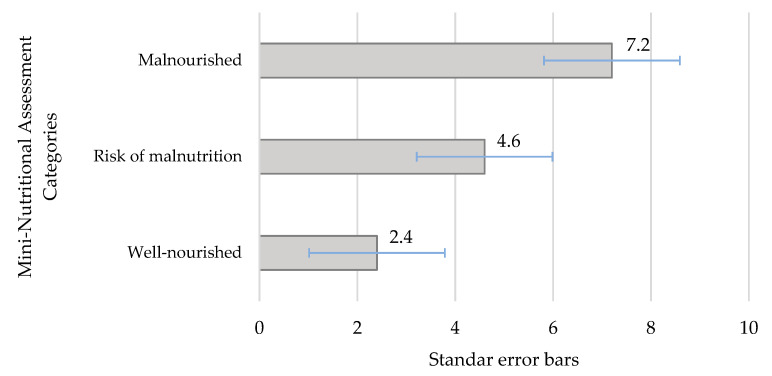
Mean of the Geriatric Depression Scale score by Mini-Nutritional Assessment categories in nursing home residents.

Figure 1 depicts the mean Geriatric Depression Scale score by Mini-Nutritional Assessment categories in the participant nursing home residents. Higher depression scores were associated with a poorer nutritional status. In fact, a negative correlation between MNA and depression was observed (*ρ* = −0.4624; *p* < 0.001). The results of the multiple logistic regression model (Table 4) indicated that residents who had depressive symptoms were approximately five times more likely to have malnutrition or be at risk of malnutrition (OR = 5.82, 95% CI = 2.27–14.89) than those who were well nourished, after adjusting for age and sex. Additionally, patients who used three or more prescription drugs per day were more likely (OR = 1.83, 95% CI = 1.27–2.63) to be malnourished or be at risk of malnutrition than their well-nourished counterparts. The interactions between sex, depressive symptoms, and use of prescription drugs were not statistically significant in the model (*p* > 0.05).

## 4. Discussion

This study confirmed and extended previous reports indicating that malnutrition is tightly associated with depression and that taking three or more prescription drugs daily is associated with a worsening nutritional status in older residents living in nursing homes [5,14,35,36]. Despite the fact that the participants evaluated resided in private nursing homes in Mexico City, malnutrition was highly prevalent. The economic conditions of these residences are precarious and sometimes it is difficult to acquire basic goods. Similar findings have been previously published; for example, in Europe, a high prevalence of poor nutrition was found in nursing home residents. In a study conducted in Germany, 57.9% of the residents were at risk of malnutrition, while 22.8% were malnourished, as determined using the MNA [37]. However, the prevalence of malnutrition varied between groups of older people despite the use of the same instrument (MNA) to evaluate the nutritional status. Several factors may explain these discrepancies, some of which are related to the specific characteristics of the various cohorts of older persons and others are related to the conditions of the residences where they lived; for example, a low food budget, large number of residents, and a shortage of staff may contribute to a poor nutritional status [22]. A systematic review that included 13 cross-sectional, one retrospective, and two prospective studies found that factors mainly associated with depression in nursing home patients were poor nutritional status with a lower BMI, impaired BI, dementia, swallowing/chewing difficulties, poor oral intake, and advanced age, as well as the presence of geriatric syndromes (fecal incontinence, falls, insomnia, psychiatric conditions, and neurological disorders) [21].

In general, malnourished patients developed weight loss in association with anorexia, loss of skeletal muscle, low anthropometric indexes, energy intake deficiency, and altered biochemical markers. Thus, it is important to identify malnutrition in older people in the face of its relationship with adverse outcomes, such as functional impairment, poor quality of life, and increased healthcare costs and mortality rates [38].

However, poor nutritional status is not only associated with depression or polypharmacy in older people. Previously published studies showed a strong association between physical dependency and poor nutritional status in Mexican nursing home residents [4]. In a cross-sectional study in community-dwelling older Chinese adults, it was found that complete physical functional dependence was strongly associated with malnutrition [39]. Additionally, in a Turkish cross-sectional study of community-dwelling older adults who received home-care services, a positive correlation was found between the BI and MNA scores; furthermore, in the multiple regression model, the BI maintained its significance after controlling for dementia, depression, and anthropometric values [40]. In the current study, most anthropometric measurements and the BI were altered in malnourished nursing home residents. Similar observations were reported in other studies, where an association between undernutrition and dependency was also found [22,37].

Our results revealed that 39.3% of the nursing home participants had depression (27.9% mild and 11.4% severe depression). Furthermore, recent widowhood was found to be a risk factor for the appearance of depressive symptoms in the participants [41]. In this regard, it was also observed that family support is very important for maintaining physical and mental health in older people [42,43].

A comparison with other studies showed a higher prevalence of depression in nursing home residents in Germany (34.7% mild and 21.3% severe depression) [37] than in Mexican residents. In a study of nursing home residents in the Netherlands, using the GDS, it was found that 46.2% of the study group had depression: subclinical depression (24.0%), minor depression (14.1%), or major depression (8.1%) [7]. An even higher prevalence of depression was found by Xie et al., who reported a rate of 74.4% in empty-nest older people living in rural areas in China [44]. In contrast, in a large prospective cohort study, also conducted in China, Cao et al. found a low prevalence [45]. Approximately one-quarter of the older people in urban areas met the GDS criterion for depression. There are several biological, psychological, and social factors that may account for the appearance of depression symptoms in patients who live in nursing homes; for example, dependence for activities of daily living, cognitive impairment, lack of social support, loneliness, exposure to stressful events, and negative subjective perception about their health [46].

In the present study, depression was higher in women than in men; these results are similar to other studied populations, such as in Chinese older people, where a higher percentage of depression was found in women [47]. Additionally, in Iranian community-dwelling older adults, depression was reported almost twice as frequently in women than in men [48]. Thus, promoting a gendered perspective in the diagnosis and treatment of depression may improve the quality of life of older women. Sociocultural factors may also influence the risk of depression by gender. Interestingly, other studies failed to establish an association between sex and depression [49].

Although depression is a common health problem in older persons living in nursing homes, this condition goes frequently undiagnosed. In fact, in the current study, more than half of the patients with depressive symptoms were previously undiagnosed. The delay in the diagnosis of depression consequently produces a delay in the medical treatment, which may affect the health of the patients, reducing their social and physical activities and their quality of life [50]. In this regard, a longitudinal study in Spain showed that the mean life expectancy increased by 1.8 years in residents in which depression was detected and treated promptly; in contrast, life expectancy decreased by 6.3 years in undetected cases [6].

In the current study, the association between malnutrition and depression was tight (OR = 5.82); likewise, in a study of German older people, the GDS score was the only factor that had a significant influence on nutritional status [37]. Furthermore, in Israel, older patients at risk of malnutrition had significantly higher GDS scores than patients with a normal nutritional status, after controlling for age, cognitive status, and functional ability [12]. Additionally, in the PEN-3S study in Portuguese elders, the MNA score was associated with depression, low appetite, and dependency in ADL [36]. In yet another study, Cabrera et al. found that the risk of developing depression was four times higher in older adults suffering from malnutrition than in those with normal nutritional status [51]. Data in the literature suggests that in several population groups around the world, there is a clear association between depression and malnutrition. Based on these findings, we believe that international organizations promoting health in older adults should take this association into account when designing their practice guidelines and programs.

Although it is not clear whether depression is a cause or an effect of malnutrition, depression is clearly a major factor affecting appetite, nutritional behavior, and dietary intake. Alterations in several neurotransmitters and hormonal changes are associated with depression. These alterations may reduce appetite [52]. Additionally, depression has been found to be a risk factor for weight loss in older people [53]. Once again, older adults are at a high risk of malnutrition, particularly if they are living in nursing homes, have few years of formal education, and take a high number of prescription drugs; all these factors facilitate the onset of the depression [37].

On average, approximately five medications were taken daily by the participants included in this study, which is similar to a study of Japanese home-care patients, where 5.9 medications were taken daily [14]. In Israel, German et al. studied hospitalized older people, and almost half of them used more than five drugs/day for the treatment of various chronic diseases [12]. In the present study, in addition to depression, the use of three or more drugs daily was associated with poor nutritional status. Similarly, in the METABOLIC Study involving Italian older patients with type 2 diabetes, an association between malnutrition and the number of drugs taken was observed. For malnourished patients, the odds of receiving five or more drugs was double that of those who were well nourished [54].

In an article based on longitudinal studies of the risk factors for malnutrition, age and frailty in institutionalized subjects, poor general health, decreased physical function, and excessive polypharmacy, among other factors, were associated with an increased risk [55]. The relationship between the number of drugs taken and nutritional status is complex; for instance, the number of drugs taken by well-nourished elderly patients in Finland was 7.7, which increased to 8.3 in the elderly individuals who were at risk of malnutrition, and decreased to 7.9 in malnourished elderly [15]. Physicians might decrease the number of drugs prescribed as the patient’s health deteriorates [14], which may be associated with geriatric failure-to-thrive syndrome [56]. 

The impact of polypharmacy on malnutrition is complex and a two-way relationship may actually be at play. Drugs could alter the taste buds and cause hyposalivation and loss of appetite, thus decreasing food intake. Conversely, poor nutritional status is associated with the development of comorbidities that will, in turn, require the use of drugs for treatment [57]. Because of this bidirectional relationship, physicians need to be careful when prescribing medications to older patients in order to avoid the unnecessary consumption of drugs and the risk of drug interactions.

The complex relationship between malnutrition, depression, and polypharmacy during aging has been explored and there is evidence of reverse causation [14,37,53,57,58,59]. Longitudinal designs and structural equation models might be helpful for analyzing these interdependent variables. 

Some strengths of our study are the high participation rate of the nursing home residents and the use of validated instruments in the assessment of nutritional status and depression with the participation of qualified professionals. 

However, one limitation of our study is its cross-sectional design, which did not allow for a causal relationship to be established. Unfortunately, a follow-up study in older home residents poses important ethical restrictions when no therapeutic interventions are provided. Another limitation is that the studied sample was selected from private nursing homes and therefore our findings may not be directly extrapolated to public nursing homes in Mexico.

## 5. Conclusions

The majority of the older adults studied were at risk of malnutrition or malnourished. Moreover, depression was highly prevalent among nursing home residents. A strong association was found between poor nutritional status and depression. Additionally, an association was observed between poor nutritional status and taking more than three drugs daily. Systematic assessment of nutritional status and depression is of utmost importance in nursing home residents.

## Figures and Tables

**Table 1:** Demographic, socio-economic, clinical, and nutritional characteristics of the study participants according to sex.

Variable	Total	Men	Women	*p*-Value
	*n* = 262	*n* = 88	*n* = 174	
Age (years), mean ± SD	83.1 ± 8.6	80.9 ± 8.6	84.2 ± 8.5	0.0036
Length of stay (years), mean ± SD	4.1 ± 4.4	3.2 ± 3.8	4.5 ± 4.6	0.0276
Formal education (years), mean ± SD	4.2 ± 2.8	4.6 ± 2.8	4.0 ± 2.7	0.1760
Marital status				
Married, *n* (%)	42 (16.0)	28 (31.8)	14 (8.1)	<0.0001 *
Widowed, *n* (%)	111 (42.4)	24 (27.3)	87 (50.0)	
Non-married/never married, *n* (%)	109 (41.6)	36 (40.9)	73 (41.9)	
Specific diseases				
Hypertension, *n* (%)	153 (58.4)	46 (52.3)	107 (61.5)	0.1537
Diabetes, *n* (%)	59 (22.5)	20 (22.7)	39 (22.4)	0.9543
Cardiovascular disease, *n* (%)	46 (17.6)	11 (12.5)	35 (20.1)	0.1173
Rheumatic disease, *n* (%)	13 (5.0)	4 (4.5)	9 (5.2)	0.8241
Thyroid disease, *n* (%)	23 (8.8)	2 (2.3)	21 (12.1)	0.0034
Renal failure, *n* (%)	11 (4.2)	4 (4.5)	7 (4.0)	0.8431
Number of drugs, mean ± SD	5.1 ± 3.9	5.2 ± 4.7	5.1 ± 3.4	0.7153
Anthropometric measurements				
Weight (kg), mean ± SD	57.6 ±12.2	63.1 ± 12.7	54.8 ± 12.6	0.0841
Height (cm), mean ± SD	153.2 ± 9.6	162.8 ± 6.9	148.3 ± 6.7	<0.0001
Body mass index (kg/m^2^), mean ± SD	24.5 ± 5.2	23.8 ± 4.3	24.9 ± 5.6	0.3532
Body mass index (<18.5 kg/m^2^), *n* (%)	28 (10.7)	11 (12.5)	17 (9.8)	0.5040
Body mass index (<22 kg/m^2^), *n* (%)	90 (34.4)	30 (34.1)	60 (34.5)	0.9497
Calf circumference (cm), mean ± SD	31.0 ± 4.3	31.7 ± 4.0	30.6 ± 4.4	0.7184
Mid-upper-arm circumference (cm), mean ± SD	26.4 ± 4.1	26.3 ± 3.6	26.4 ± 4.3	0.9704
Tricipital skinfold (mm), mean ± SD	14.4 ± 6.7	11.9 ± 5.7	15.6 ± 6.9	0.0413
Arm muscle circumference (cm) mean ± SD	21.9 ± 3.2	22.6 ± 2.9	21.5 ± 3.3	0.0869
Mini Nutritional Assessment (MNA)				
MNA (points)	19.9 ± 4.1	20.7 ± 3.8	19.5 ± 4.2	0.0570
MNA Classification				
Well-nourished, *n* (%)	47 (17.9)	19 (21.6)	28 (16.1)	0.0783 **
At risk of malnutrition, *n* (%)	157(59.9)	55 (62.5)	102 (58.6)	
Malnourished, *n* (%)	58 (22.1)	14 (15.9)	44 (25.3)	
Geriatric Depression Scale (GDS)				
GDS (points), mean ± SD	4.8 ± 3.5	4.1 ± 3.0	5.1 ± 3.6	0.0275
Normal, *n* (%)	159 (60.7)	57 (64.8)	102 (58.6)	0.0149 ***
Mild depression, *n* (%)	73 (27.9)	28 (31.8)	45 (25.9)	
Severe depression, *n* (%)	30 (11.4)	3 (3.4)	27 (15.5)	
Mild and severe depression, *n* (%)	103 (39.3)	31 (35.2)	72 (41.4)	
Barthel Index (BI)				
BI (points)	65.0 ± 35.0	69.7 ± 32.3	62.5 ± 36.1	0.0133
Independent, *n* (%)	67 (25.6)	25 (28.4)	42 (24.1)	0.5452 ****
Mildly dependent, *n* (%)	25 (9.5)	8 (9.1)	17 (9.8)	
Moderately dependent, *n* (%)	66 (25.2)	24 (27.3)	42 (24.1)	
Severely dependent, *n* (%)	50 (19.1)	18 (20.4)	32 (18.4)	
Totally dependent, *n* (%)	54 (20.6)	13 (14.8)	41 (23.6)	

* p-value for the *χ*^2^ test for categories of marital status, ** *p*-value for the *χ*^2^ test for the MNA classification categories, *** *p*-value for the *χ*^2^ test for categories of the Geriatric Depression scale, **** *p*-value for the *χ*^2^ test for categories of the Barthel Index.

**Table 2:** Anthropometric characteristics, Geriatric Depression Scale (GDS), Barthel Index (BI), nursing home length of stay, and the number of drugs used by nutritional status.

Variable	Total (*n* = 262)				
	Normal Nutrition *n* = 47 Mean ± SD	Risk of Undernutrition *n* = 157 Mean ± SD	*p* *	Undernutrition *n* = 58 Mean ± SD	*p* **
Age (years)	80.6 ± 8.1	82.8 ± 8.4	<0.001	86.0 ± 9.3	<0.001
Weight (kg)	66.7 ± 13.3	57.8 ± 12.2	<0.001	49.5 ± 10.9	<0.001
Height (cm)	154.9 ± 10.9	152.7 ± 9.7	0.795	153.1 ± 8.3	0.398
Body mass index (kg/m^2^)	27.9 ± 5.4	24.8 ± 4.6	<0.001	21.2 ± 4.5	<0.001
Calf circumference (cm)	34.1 ± 3.3	30.8 ± 5.3	0.236	27.8 ± 3.7	0.002
Mid-upper-arm circumference (cm)	29.3 ± 3.6	26.5 ± 3.7	<0.001	23.6 ± 3.7	<0.001
Tricipital skinfold (mm)	17.6 ± 6.9	14.4 ± 6.6	<0.001	11.3 ± 5.9	<0.001
Mid-upper-arm muscle circumference (cm)	23.7 ± 2.9	22.0 ± 3.0	<0.001	20.0 ± 3.2	<0.001
GDS (score)	2.4 ± 2.2	4.6 ± 3.1	<0.001	7.2 ± 3.7	<0.001
BI (points)	92.1 ± 14.1	68.1 ± 32.7	<0.001	34.2 ± 30.3	<0.001
Length of stay (years)	3.8 ± 4.0	3.9 ± 4.0	0.479	4.8 ± 5.6	0.388
Number of drugs	4.9 ± 4.4	5.3 ± 3.9	0.557	4.8 ± 3.7	0.915

* *p*-value of the multinomial logistic regression comparing normal with the risk of undernourished nursing home residents. ** *p*-value of the multinomial logistic regression comparing normal with the undernourished nursing home residents.

**Table 3:** Characteristics according to evaluation by the Geriatric Depression Scale.

Variable	Total (*n* = 262)		
	Normal *n* = 159 Mean ± SD	Mild and Severe Depression *n* = 103 Mean ± SD	*p* *
Age (years)	82.7 ± 8.7	83.7 ± 8.7	0.295
Weight (kg)	58.2 ± 13.3	56.6 ± 13.2	0.184
Height (cm)	153.7 ± 10.0	152.4 ± 9.1	0.181
Body mass index (kg/m^2^)	24.6 ± 5.0	24.4 ± 5.5	0.577
Calf circumference (cm)	31.6 ± 4.4	29.3 ± 5.8	0.001
Mid-upper-arm circumference (cm)	26.7 ± 4.1	25.9 ± 4.1	0.021
Tricipital skinfold (mm)	14.9 ± 6.8	13.3 ± 6.8	0.001
Mid-upper-arm muscle circumference (cm)	22.0 ± 3.2	21.7 ± 3.2	0.442
MNA (points)	21.2 ± 3.7	17.9 ± 4.0	0.001
BI (points)	73.1 ± 13.5	52.3 ± 36.5	0.001
Length of stay (years)	4.3 ± 4.6	3.7 ± 4.1	0.174
Number of drugs	5.3 ± 4.1	4.8 ± 3.6	0.053

* *p*-value of the Kruskal–Wallis test.

**Table 4:** Odds ratios of malnutrition or be at risk of malnutrition and sociodemographic variables, number of drugs used, and depression among aged residents in nursing homes.

Variable	Crude	OR 95% CI	*p*	Adjusted ^1^	OR 95% CI	*p*
Age	1.04	1.01–1.08	0.034	1.03	1.01–1.06	0.004
Sex (female) ^2^	1.53	0.80–2.93	0.198	1.28	0.56–2.89	0.564
Drugs (≥3/day) ^3^	1.71	1.22–2.40	0.002	1.83	1.27–2.63	<0.001
Depression (GDS > 5) ^4^	5.80	2.22–15.12	<0.001	5.82	2.27–14.89	<0.001

^1^ Robust estimates, standard errors adjusted for three clusters (nursing homes). ^2^ Reference categories sex: male, ^3^ polypharmacy <3 drugs/day, ^4^ Geriatric Depression Scale (GDS) ≤5.
